# Epicardial placement of human placental membrane allografts in coronary artery bypass graft surgery is associated with reduced postoperative atrial fibrillation: a pilot study for a future multi-center randomized controlled trial

**DOI:** 10.1186/s13019-024-02822-8

**Published:** 2024-06-01

**Authors:** Zain Khalpey, Ujjawal Kumar, Pamela Hitscherich, Usman Aslam, Evangelia Chnari, Marc Long

**Affiliations:** 1 Department of Cardiothoracic Surgery, Heart and Vascular Institute, 10210 N 92nd St Suite 300, HonorHealth, Scottsdale, AZ 85258 USA; 2 Gonville & Caius College, University of Cambridge, Trinity Street, Cambridge, CB2 1TA UK; 3 MTF Biologics, 125 May Street, Edison, NJ 08837 USA; 4 General Surgery Residency Program, HonorHealth, Phoenix, AZ 85250 USA

**Keywords:** Post-operative atrial fibrillation, Coronary artery bypass grafting, Aseptically processed human placental membrane allograft.

## Abstract

**Background:**

Post-operative atrial fibrillation (POAF) occurs in up to 40% of patients following coronary artery bypass grafting (CABG) and is associated with a higher risk of stroke and mortality. This study investigates how POAF may be mitigated by epicardial placement of aseptically processed human placental membrane allografts (HPMAs) before pericardial closure in CABG surgery. This study was conducted as a pilot feasibility study to collect preliminary for a forthcoming multi-center randomized controlled trial.

**Methods:**

This retrospective observational study of patients undergoing CABG surgery excluded patients with pre-operative heart failure, chronic kidney disease, or a history of atrial fibrillation. The “treatment” group (*n* = 24) had three HPMAs placed epicardially following cardiopulmonary bypass decannulation but before partial pericardial approximation and chest closure. The only difference in clinical protocol for the control group (*n* = 54) was that they did not receive HPMA.

**Results:**

HPMA-treated patients saw a significant, greater than four-fold reduction in POAF incidence compared to controls (35.2–8.3%, *p* = 0.0136). Univariate analysis demonstrated that HPMA treatment was associated with an 83% reduction in POAF (OR = 0.17, *p* = 0.0248). Multivariable analysis yielded similar results (OR = 0.07, *p* = 0.0156) after controlling for other covariates. Overall length of stay (LOS) between groups was similar, but ICU LOS trended lower with HPMA treatment (*p* = 0.0677). Post-operative inotrope and vasopressor requirements were similar among groups. There was no new-onset post-operative heart failure, stroke, or death reported up to thirty days in either group.

**Conclusions:**

Epicardial HPMA placement can be a simple intervention at the end of CABG surgery that may provide a new approach to reduce post-operative atrial fibrillation by modulating local inflammation, possibly reducing ICU and hospital stay, and ultimately improving patient outcomes.

## Background

Heart disease, including coronary artery disease, remains the leading cause of death in the United States [1]. Despite advances in guideline-directed medical therapies, as well as percutaneous interventions, coronary artery bypass grafting (CABG) remains the gold standard treatment for many cases of coronary artery disease. It is therefore one of the most commonly performed surgical procedures, with over 200,000 in the US and almost 800,000 worldwide per year [2]. Up to 40% of patients who undergo CABG surgery will experience new-onset postoperative atrial fibrillation (POAF) [3, 4], typically occurring in the first five postoperative days [5]. Patients who develop POAF are at higher risk of inpatient mortality, extended admission, and increased risk of major adverse cardiovascular events (MACE), i.e., stroke and cardiac-related death [6]. Despite POAF resolving in about 90% of patients at 8 weeks post-operatively [7], POAF remains a significant independent predictor of long-term mortality [8]. Additionally, POAF confers a financial and operational burden on hospitals performing cardiac surgery, as well as health systems as a whole. The financial burden of POAF was shown clearly in a randomized trial comparing on and off-pump cardiac surgery, that demonstrated the cost difference at 1 year for patients who developed POAF was $15,593 more than those who did not, due to longer hospital admissions and complications of POAF [9]. Current clinical approaches to reduce new-onset postoperative atrial fibrillation include medications such as 𝛽-blockers and amiodarone. However, since the exact pathophysiology of POAF remains to be understood [10], it is difficult to develop a pathophysiology-targeted approach. However, the time course of POAF, occurring within the first 5 days of surgery corresponds to a higher level of inflammatory biomarkers [11], such as the advanced glycation end products (AGE) pathway and reactive oxygen species (ROS), linked with high levels of epicardial and pericardial inflammation on post-operative days 3–6 [12]. Additionally, some anti-inflammatory agents have shown some early promise in reducing POAF incidence [13] with early studies of placental tissue in cardiovascular applications also showing success [14].

Placental tissue is known to be immunologically privileged, containing numerous growth factors and cytokines involved in cell behavior, inflammation, and angiogenesis [15]. Consequently, it has been used successfully as a wound covering in plastic surgery and burns treatment, for example, offering anti-inflammatory properties that foster more organized wound healing [16, 17]. The cardiac inflammation resulting from CABG surgery is likely to benefit similarly and localized application of placental membrane may reduce post-operative complications associated with disorganized healing and inflammation, including POAF. This study aimed to test the hypothesis that aseptically processed human placental membrane allograft (HPMA) placement as an epicardial tissue covering during CABG procedures would reduce POAF incidence.

## Methods

This is a retrospective observational study, comprised of seventy-eight patients who underwent coronary artery bypass grafting surgery performed by a single surgeon between 2019 and 2021. Institutional Review Board ethical approval was granted for outcome analysis in this study (IRB#20200195) and informed consent was obtained from all patients for the relevant surgical procedures as well as for analysis of anonymized data in this study. All methods of this study were conducted per the relevant guidelines and regulations for working with human subjects. All patients included were over the age of 18. Exclusion criteria were pre-existing diagnoses of heart failure (HF), chronic kidney disease (CKD), or a history of atrial fibrillation (AFib). Heart failure was defined using American Heart Association (AHA) criteria for diastolic heart failure [18] – left ventricular ejection fraction (LVEF) less than or equal to 40% as measured with transthoracic echocardiography. Chronic kidney disease was defined using the internationally accepted Kidney Disease: Improving Global Outcomes (KDIGO) criteria [19] – glomerular filtration rate less than 60 ml/min/1.73m^2^ and albuminuria with an albumin-creatinine ratio greater than 3 mg/mmol.

### Data collection

Pre-operative demographic (age and gender) and clinical data of all patients were collected from the institutional electronic medical record system [20]. Clinical data were classified as either cardiac characteristics (pre-operative LVEF, Society of Thoracic Surgeons (STS) risk score, or a clinical history of myocardial infarction (MI) by ICD-10 code, coronary stenting, or 𝛽-blocker usage) or non-cardiac characteristics (height, weight, body mass index (BMI), or a clinical history of hypertension or diabetes mellitus by ICD-10 code). Additional noncardiac characteristics included were renal function indices, glomerular filtration rate, and serum creatinine, from a fasting blood sample obtained on the last pre-operative morning. Perioperative data was collected: the number of vessels bypassed in the CABG procedure as well as total cardiopulmonary bypass (CPB) and aortic cross-clamp time (XCT). Additionally, multiple post-operative outcome parameters were collected: new-onset postoperative atrial fibrillation incidence and time to onset, amiodarone and colchicine requirement for arrhythmia treatment, post-operative LVEF, post-operative inotropic and pressor requirement, duration and type, as well as post-operative acute kidney injury (AKI) incidence and time to onset. Lastly, data was collected for length of total hospital and intensive care unit (ICU) admission, as well as incidence of new-onset heart failure and thirty-day incidence of stroke and death. AKI was defined using KDIGO criteria [21].

New-onset postoperative atrial fibrillation was defined using the Society of Thoracic Surgeons (STS) criteria [22]. In order to detect postoperative atrial fibrillation while the patient is admitted to the hospital postoperatively, we use a three- pronged diagnostic strategy. Firstly, patients are kept on continuous, real-time telemetry electrocardiogram (EKG) monitoring from the time they leave the operating room until they are discharged home. If the telemetry monitoring detects atrial fibrillation, a confirmatory 12-lead EKG is carried out within five minutes to assess cardiac rhythm. Lastly, the physician caring for the patient is also alerted at the same time, and they confirm the rhythm to be atrial fibrillation. We recognize that varying definitions of postoperative atrial fibrillation exist, hence in our clinical practice, we exclusively use the STS criteria, which are outlined below:

1. Duration: The atrial fibrillation or flutter episode should last for at least 30 s.
2. Timing: The episode should occur during the postoperative period, which is typically defined as the time from the end of the surgical procedure until 30 days after the surgery.
3. Documentation: The atrial fibrillation or flutter should be documented by a 12-lead electrocardiogram (ECG) or a rhythm strip, confirming the diagnosis. AF should be documented by ECG, with irregular RR intervals and an absence of distinct P waves, lasting for at least 30 s or for the duration of the ECG recording if less than 30 s.
4. Exclusion of pre-existing atrial fibrillation: Patients with a history of atrial fibrillation or flutter before the surgery are not considered to have POAF unless they were in stable sinus rhythm for at least 3 months before the surgical procedure.
5. Exclusion of secondary causes: Other potential causes of AF, such as electrolyte imbalances, thyroid disorders, or acute infections, should be ruled out or treated accordingly.

### Operative technique and clinical protocol

**Fig. 1:**
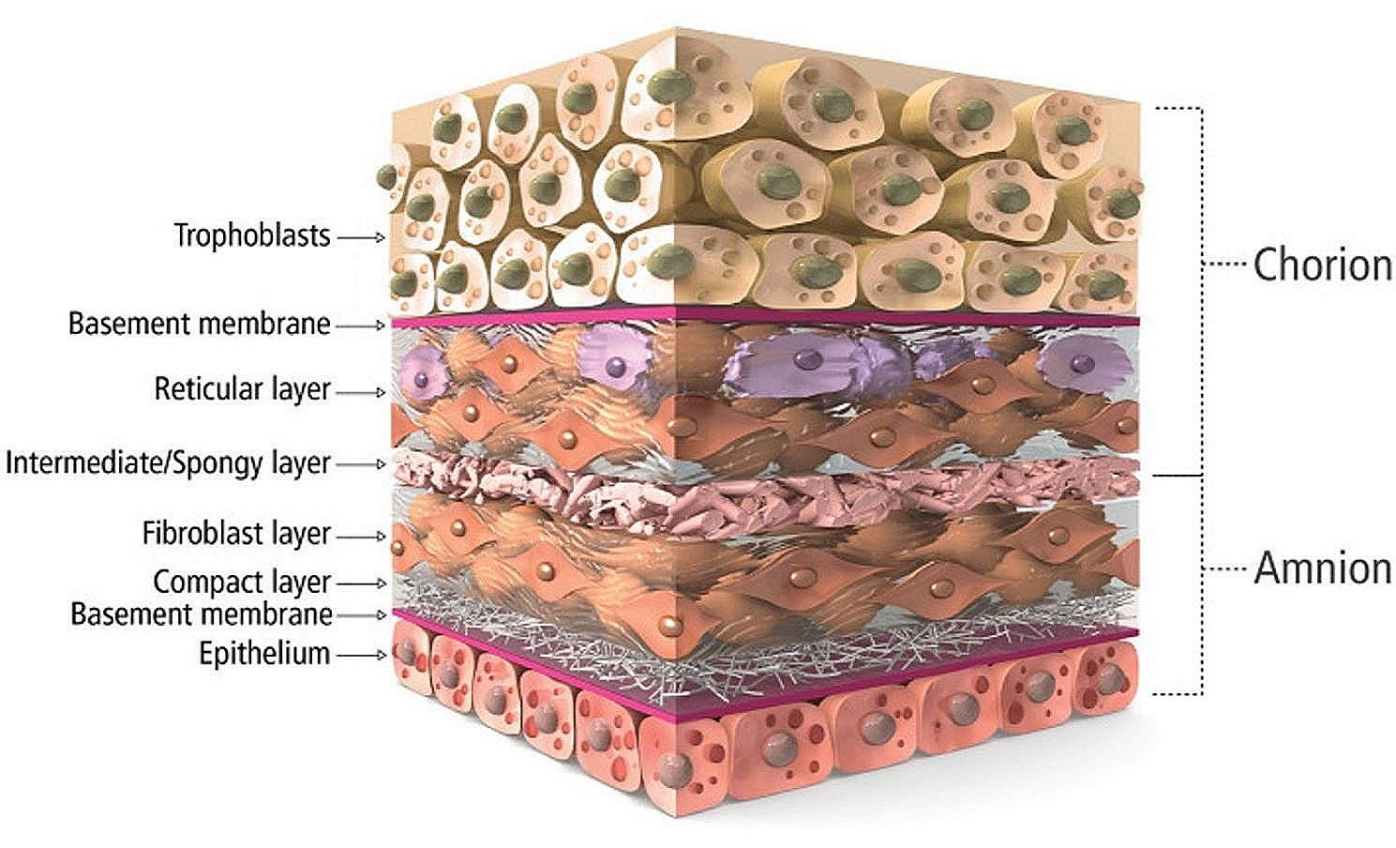
The HPMAs used were comprised of both placenta-derived amnion and chorion, superior to amnion-only membranes, by improving handling properties due to a thicker tissue bilayer, as well as an increased amount of naturally preserved placental factors

**Fig. 2:**
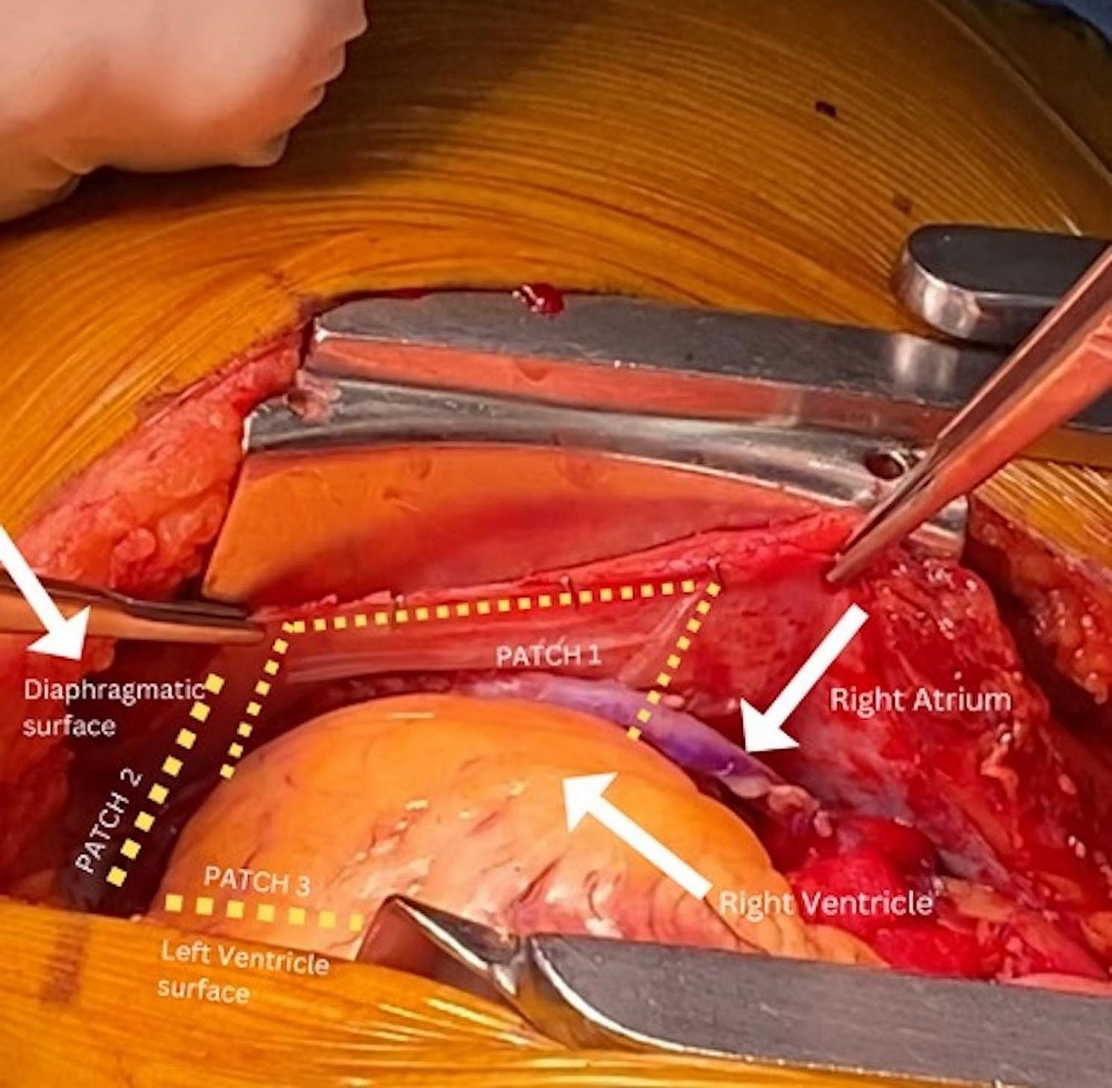
Placement of 3 HPMAs (5 × 6 cm) prior to pericardial approximation and closure

The HPMAs used in this study (MTF AmnioBand® Matrix, MTF Biologics, Edison, NJ) are aseptically processed amnion and chorion tissue forms regulated for use as a Human Cellular and Tissue-Based Product (HCT/P) under FDA 21 CFR 1271 and Sect. 361 of the Public Health Service (PHS) Act when labeled and promoted for use in wound treatment and management as a protective covering for internal or external soft tissue defects including acute, chronic, and surgically created wounds. Three HPMAs were placed over the epicardium once the patient had been weaned off cardiopulmonary bypass and had been decannulated in preparation for partial pericardial approximation and chest closure. Each patch measured 5 cm x 6 cm, with a schematic representation shown in Fig. 1). The patches were placed as follows, and as depicted in Fig. 2:

- Right atrium: secured with two medium clips to the pericardium which was then approximated over the right atrium.
- Right ventricle: secured inferiorly with two medium clips to the diaphragmatic surface.
- Left ventricle: placed topically and the pericardium approximated over the left ventricular membrane.

This placement strategy was employed to minimize movement of the membranes due to the beating heart. Twenty-four patients were treated with HPMA (treatment group) and were compared to fifty-four non-HPMA patients (control group), where the only procedural difference was the lack of HPMA placement.

Additionally, all patients in this study underwent left atrial appendage ligation (LAAL) with a 40 mm clip (AtriClip, AtriCure Inc, Mason, OH, USA), which was placed atraumatically to the base of the left atrial appendage (LAA), and a residual stump of less than 0.5 cm was confirmed using multiple angles on transesophageal echocardiography. Work from our group has shown that in a large group of similar patients undergoing isolated CABG surgery as in this study, carrying out successful left atrial appendage closure (with TEE confirmation and good LVEF postoperatively) eliminates the need for anticoagulation postoperatively even if new-onset AFib (POAF) develops [23]. Hence, patients were not formally anticoagulated even if they developed postoperative AFib.

All patients in this study, regardless of study group, underwent partial pericardial reapproximation and closure with silk sutures before chest closure. All patients were administered POAF prophylaxis in the form of beta-blockers; if a patient was not on “long-term” beta-blockers preoperatively, they were started on admission to the hospital before surgery and were continued in all patients postoperatively.

### Post-operative follow-up

Follow-up appointments in the outpatient setting were scheduled at two weeks and thirty days following hospital discharge after the initial procedure. A detailed multi-system clinical evaluation and physical exam were performed at each visit. Thirty-day all-cause mortality and stroke incidence were also evaluated at follow-up. To assess for POAF following hospital discharge, every patient would have a 12-lead EKG at discharge and follow-up appointments following discharge at 14 days and 30 days. If at any point after discharge, the patient felt symptoms of AFib such as palpitations, they would be seen as soon as possible that day in the clinic and would have an urgent EKG done to assess heart rhythm.

### Statistical analysis

Data were summarized using descriptive statistics. For continuous variables, mean and standard deviation (SD) were presented for normally distributed (parametric) data while median and interquartile range (IQR) were used for non-normally (non-parametric) distributed data. We compared group differences between HPMA-treated and non-HPMA-treated patients as well as POAF and non-POAF patients. Continuous variables were compared using unpaired t-tests (parametric variables) or Mann-Whitney U tests (non-parametric variables) and categorical variables were compared using Chi-square (for frequency cells > 5) or Fisher exact (for frequency cells < 5) tests. Both univariate and multivariable regression models were conducted to examine the likelihood of POAF association with HPMA treatment using unadjusted (uOR) and adjusted (aOR) odds ratios with 95% confidence intervals (CI). All statistical analyses were performed using SAS 9.4 (SAS Inc., Cary, NC) and a p-value less than 0.05 was considered significant as is conventional.

## Results

**Fig. 3:**
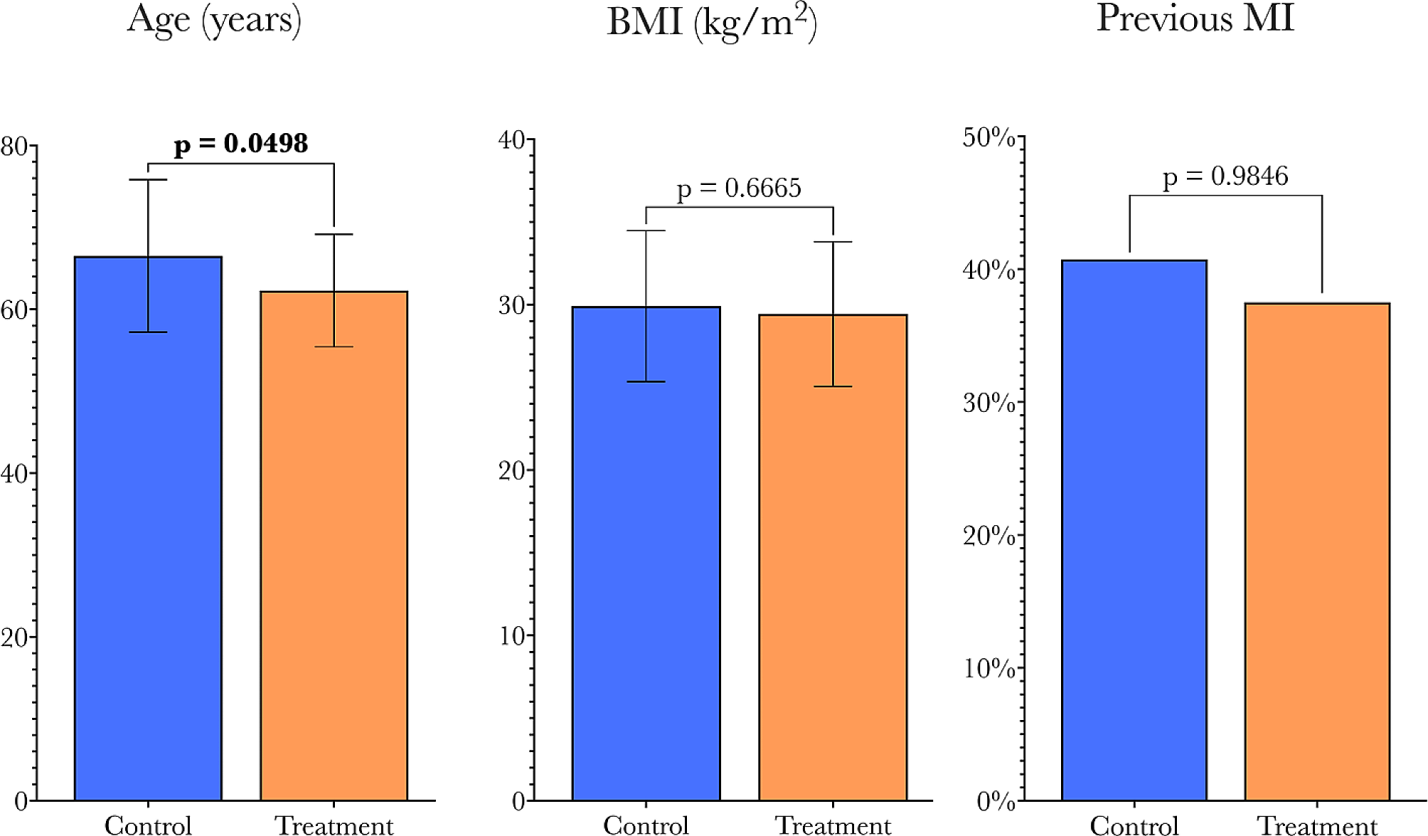
Comparison of key pre-operative risk factors for AFib (age, BMI, and history of MI) between control and treatment groups. Full comparison of characteristics is given in Table 1

Patient characteristics, stratified by study cohort, are summarized in Table 1. When comparing the HPMA-treated group to the control group, the treatment group was slightly younger (mean age = 62.3y vs. 66.5y, *p* = 0.0498), with no significant differences observed in BMI or the proportion of patients with prior MI between the two groups (Fig. 3). These characteristics are known to be risk factors for the development of POAF [24, 25].

**Table 1:** Pre-operative and operative patient characteristics by group. Categorical variables are expressed as N (%), with continuous variables expressed as either Mean ± SEM for parametric variables or as Median (IQR) for nonparametric variables

Variable	Control	Treatment	*p*-value
Number	54 (69.2)	24 (30.8)	
*Demographics*			
Age, y	66.5 ± 9.3	62.3 ± 6.9	0.0498
Gender, Male	37 (68.5)	19 (79.2)	0.3348
*Cardiac Characteristics*			
Pre-operative LVEF, %	55 (50–60)	60 (55–60)	0.0615
STS risk score, %	0.814 (0.577–1.172)	0.513 (0.398–0.868)	0.0217
Prior myocardial infarction	22 (40.7)	9 (37.5)	0.7872
Prior stenting	11 (20.4)	5 (20.8)	0.9627
Pre-operative 𝛽-blocker use	22 (40.7)	17 (70.8)	0.0142
*Non-Cardiac Characteristics*			
Body mass index, kg/m^2^	29.9 ± 4.6	29.4 ± 4.4	0.6665
Glomerular filtration rate, ml/min	81.6 ± 16.7	80.5 ± 18.3	0.7857
Pre-operative creatinine, mg/dL	0.9 (0.8–1.0)	1.0 (0.9–1.0)	0.1697
Hypertension	42 (77.8)	16 (66.7)	0.2996
Diabetes mellitus	19 (35.2)	12 (50.0)	0.2172
*Operative Characteristics*			
Number of vessels bypassed			0.5004
1	1 (1.9)	0 (0.0)	
2	15 (27.8)	6 (25.0)	
3	33 (61.1)	13 (54.2)	
4	5 (9.3)	5 (20.8)	
Cardiopulmonary bypass time, min	94.6 ± 27.9	96.0 ± 23.3	0.8342
Aortic cross-clamp time, min	67.3 ± 23.3	65.9 ± 18.2	0.7943

According to the complete STS risk score, the treatment group is at a lower risk of operative mortality. With the sample sizes involved, it would be difficult to perfectly match study cohorts, and we therefore undertook suitable statistical analyses (univariate and multivariable) to try and account for any confounding factors such as differences in STS risk score. It must be noted, additionally, that while the STS score is a good indicator of operative risk, there is not strong evidence that it is a good predictor of POAF incidence. It is also worth noting that with mean risk scores of 0.513% and 0.814% respectively (*p* = 0.0217), both the treatment and control groups consisted of relatively low operative risk candidates.

The treatment group had a significantly higher proportion of patients taking 𝛽-blockers pre-operatively (70.8% vs. 40.7%, *p* = 0.0142). However, since there was no significant difference in the incidence of hypertension between groups, and all patients with a history of AFib were excluded from this study (any undiagnosed AFib would have been discovered on pre-operative electrocardiogram), it is unlikely that this difference in pre-operative 𝛽-blocker requirement is due to any factor that may have contributed to the outcomes and therefore the results of this study. Additionally, since it is well known that 𝛽-blockers can reduce the incidence of postoperative atrial fibrillation, any patients that were not on “long-term” 𝛽-blockers preoperatively were given 𝛽-blockers perioperatively. All patients received 𝛽-blockers postoperatively to achieve pharmacological POAF prophylaxis.

**Fig. 4:**
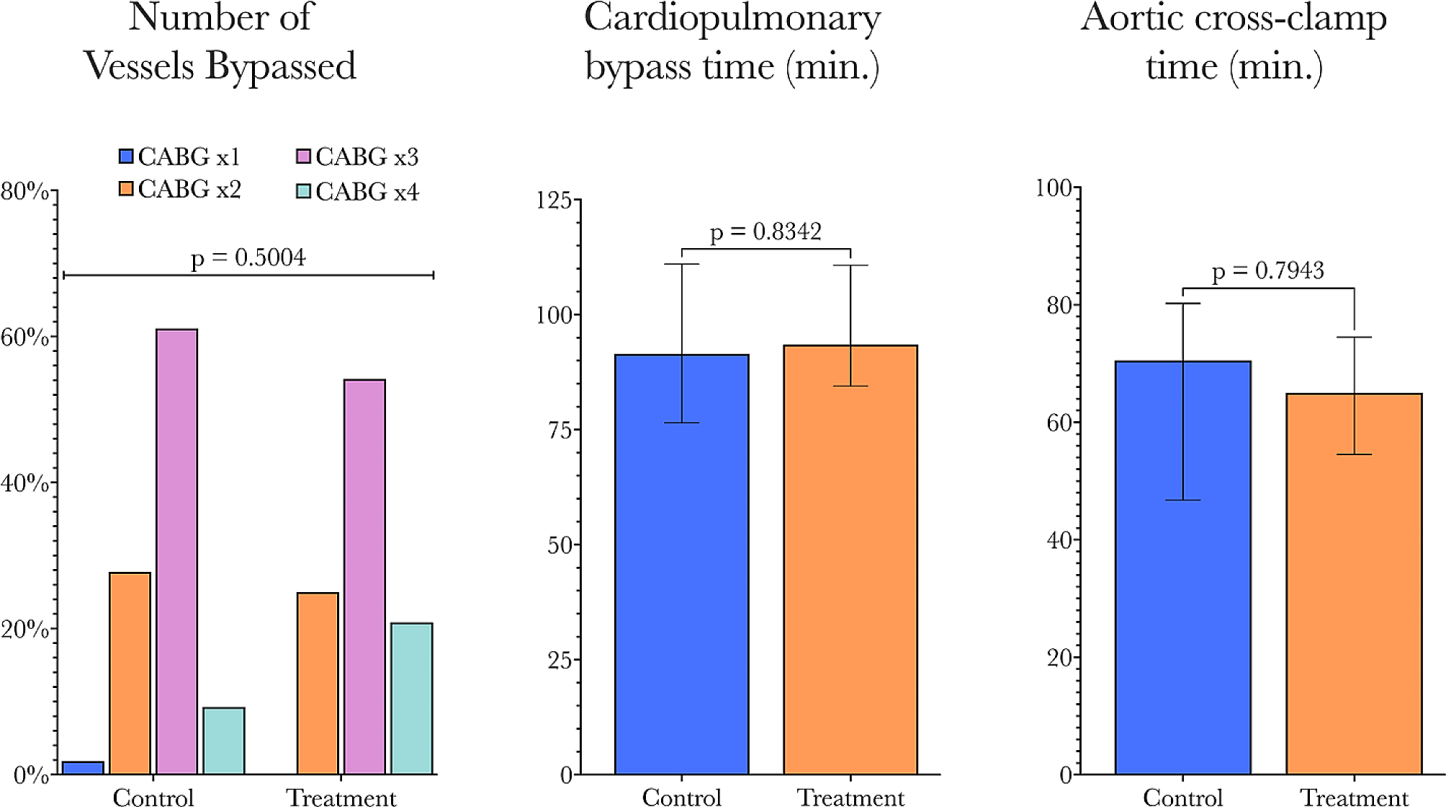
Comparison of operative parameters (number of vessels bypassed, total cardiopulmonary bypass, and aortic cross-clamp time) between control and treatment groups

Due to the non-randomized nature of this study, it was not feasible to match the demographics of the two study cohorts, and therefore these demographic differences had to be accounted for in the statistical analyses. This was achieved by undertaking both univariate and multivariable analyses, the findings of which will subsequently be discussed in detail. There were no other significant differences between HPMA-treated and non-HPMA-treated patients in the other pre-operative characteristics or operative characteristics. Similar numbers of vessels were bypassed with comparable CPB and XCT between groups. A comparison of operative characteristics is shown in Fig. 4.

We further compared sample characteristics between patients with and without POAF; the results are shown in Table 2. There were no significant differences in pre-operative and operative characteristics between patient populations that experienced POAF compared to those who did not.

**Table 2:** Pre-operative and operative patient characteristics by POAF. Categorical variables are expressed as N (%), with continuous variables expressed as either Mean ± SEM for parametric variables or as Median (IQR) for nonparametric variables

Variable	No POAF	POAF	*p*-value
Number	57 (73.1)	21 (26.9)	
*Demographics*			
Age, y	65.3 ± 8.9	65.1 ± 8.8	0.9411
Gender, Male	39 (68.4)	17 (81.0)	0.2753
*Cardiac Characteristics*			
Pre-operative LVEF, %	55 (55–60)	55 (50–60)	0.9666
STS risk score, %	0.747 (0.491–1.055)	0.639 (0.417–1.021)	0.5965
Prior myocardial infarction	22 (38.6)	9 (42.9)	0.7331
Prior stenting	11 (19.3)	5 (23.8)	0.6616
Pre-operative 𝛽-blocker use	30 (52.6)	9 (42.9)	0.4438
*Non-Cardiac Characteristics*			
Body mass index, kg/m2	29.8 ± 4.3	29.7 ± 5.2	0.9771
Glomerular filtration rate, ml/min	80.9 ± 16.4	82.3 ± 19.3	0.7558
Pre-operative creatinine, mg/dL	0.9 (0.8–1.0)	0.9 (0.8–1.0)	0.7751
Hypertension	42 (73.7)	16 (76.2)	0.8221
Diabetes mellitus	24 (42.1)	7 (33.3)	0.4826
*Operative Characteristics*			
Number of vessels bypassed			0.8003
1	1 (1.8)	0 (0.0)	
2	16 (28.1)	5 (23.8)	
3	32 (56.1)	14 (66.7)	
4	8 [14]	2 (9.5)	
Cardiopulmonary bypass time, min	94.7 ± 27.6	96 ± 23.5	0.8488
Aortic cross-clamp time, min	66.3 ± 21.0	68.2 ± 24.1	0.7336

**Fig. 5:**
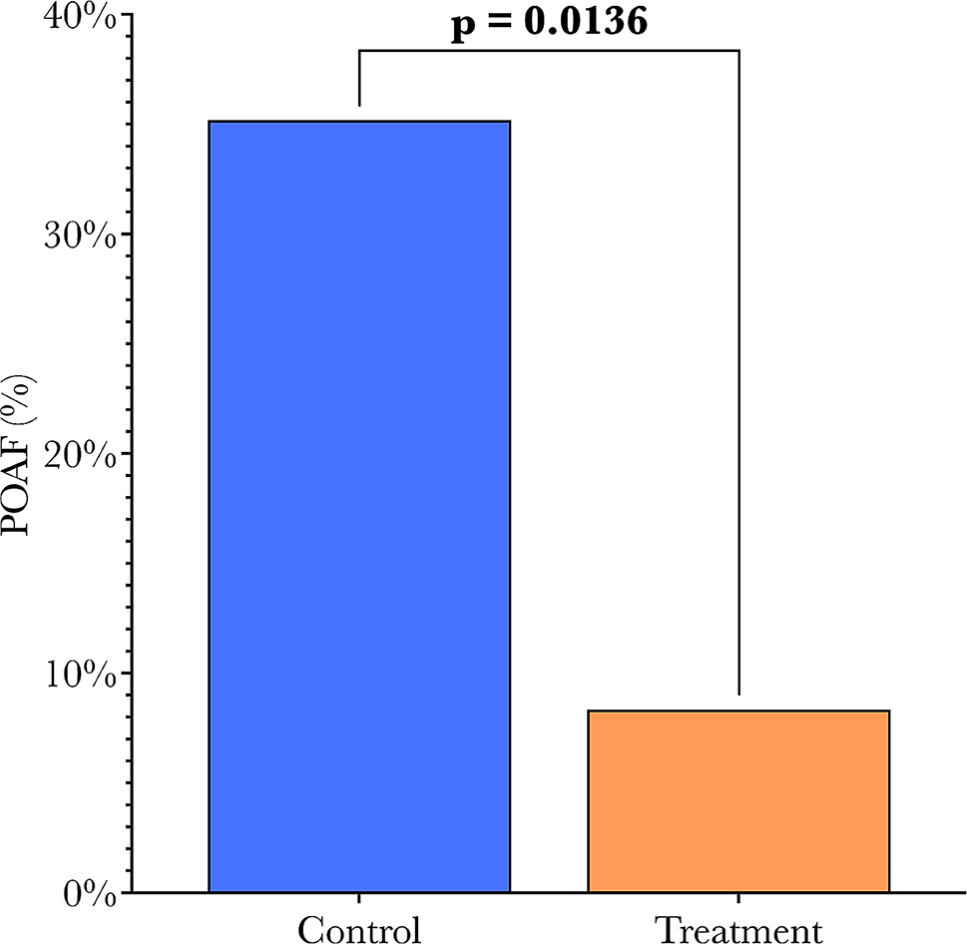
Significant decrease (*p* = 0.0136) in new onset POAF with HPMA treatment from 35.2 to 8.3%

Patients treated with HPMA saw a greater than four-fold reduction in POAF compared to control patients (8.3% vs. 35.2%, *p* = 0.0136), illustrated in Fig. 5. Although postoperative inotropic support requirement was similar between groups, control patients tended to require treatment for a longer amount of time (*p* = 0.0594). Additionally, the inotrope of choice differed significantly between groups; a greater proportion of patients in the control group were prescribed dobutamine for inotropic support, whereas patients in the treatment group tended to have norepinephrine, used as Levophed (*p* = 0.0374). While norepinephrine is typically used for its vasopressor effects, at the doses that it was used in this study, it has a greater contribution to positive inotropy than it does to vasoconstriction. While the arrhythmogenic effects of dobutamine are known, these are typically only seen at higher “cardiac” doses, around 10 micrograms/kg/minute. However, our patients received a much lower dose, up to a maximum of 2.5 micrograms/kg/minute, to prevent a risk of arrhythmias due to the dobutamine administration.

**Fig. 6:**
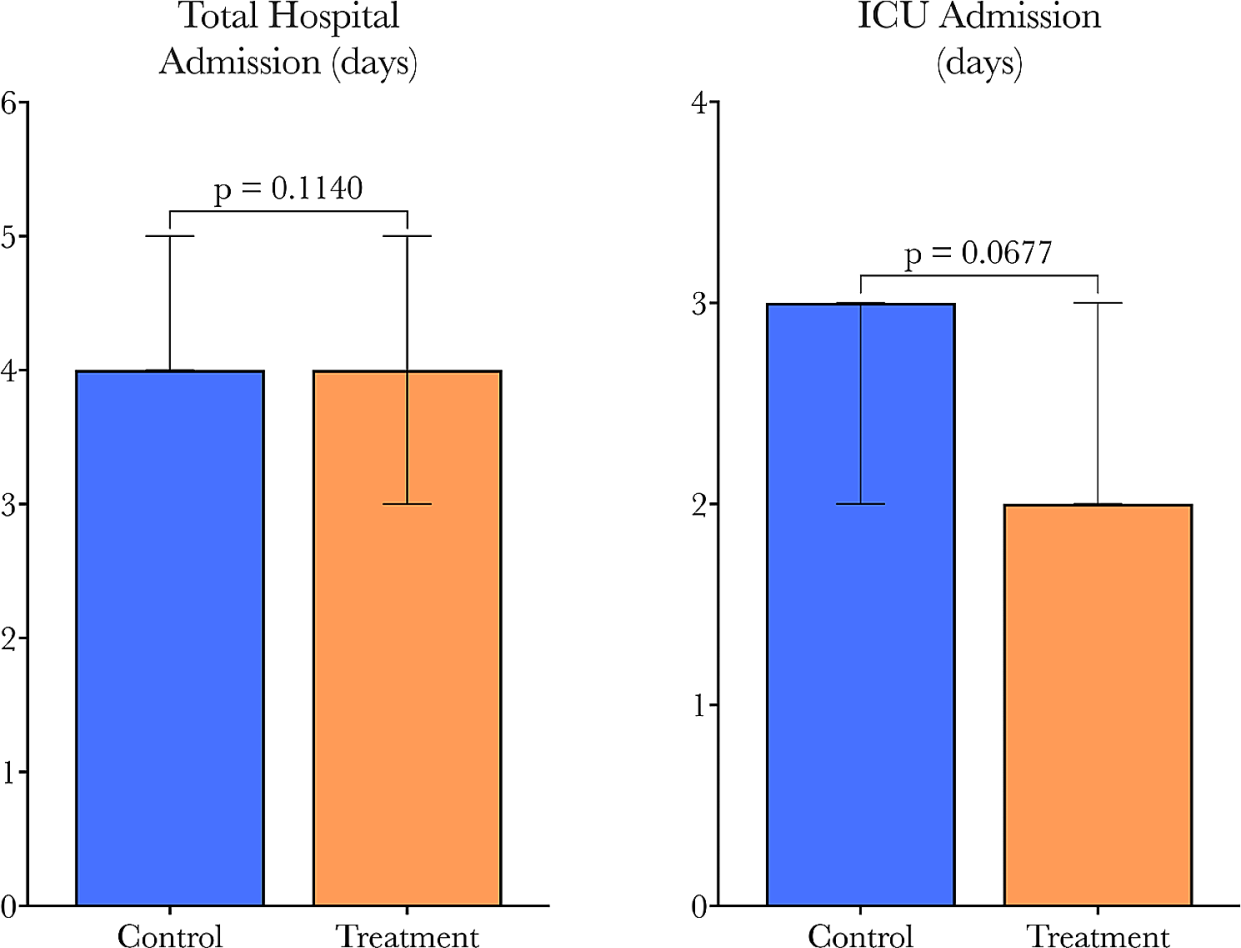
Comparison of hospital and ICU length of stay by group. No significant difference between groups, but ICU LOS trended shorter in the treatment group

While there was no significant difference in overall hospital length of stay between groups, the median ICU length of stay trended lower in patients treated with HPMA (2 vs. 3 days, *p* = 0.0677), shown in Fig. 6. With the sample sizes in this pilot study, we did not observe a statistically significant difference in the length of ICU and hospital admission, however, with a larger sample size this may be noticeable.

Incidence of postoperative AKI was similar among groups and there was no post-operative HF, stroke, or death reported up to thirty days. There were no other significant differences in the other post-operative outcomes, with all post-operative outcomes shown in Table 3.

**Table 3:** Post-operative outcomes by group. Categorical variables are expressed as N (%), with continuous variables expressed as Median (IQR).

Variable	Control	Treatment	*p*-value
Number	54 (69.2)	24 (30.8)	
POAF	19 (35.2)	2 (8.3)	**0.0136**
Days to POAF onset			0.6427
1	0 (0.0)	0 (0.0)	
2	5 (26.3)	1 (50.0)	
3	9 (47.4)	1 (50.0)	
4	5 (26.3)	0 (0.0)	
Amiodarone	12 (22.2)	2 (8.3)	0.2048
Colchicine	6 (25.0)	11 (20.4)	0.6476
Post-operative LVEF, %	55.0 (55.0–60.0)	60.0 (55.0–60.0)	0.2126
Post-operative inotropic support	14 (25.9)	5 (20.8)	0.6287
Type of inotropic support			0.0374
Dobutamine	13 (92.9)	2 (40.0)	
Norepinephrine	1 (7.1)	3 (60.0)	
Days of inotropic support			0.0594
1	3 (21.4)	4 (80.0)	
2	7 (50.0)	1 (20.0)	
3	4 (28.6)	0 (0.0)	
Pressor support	10 (18.5)	4 (16.7)	0.8441
Post-operative AKI	12 (22.2)	5 (20.8)	0.8909
Days to AKI			0.4682
1	4 (33.3)	3 (60.0)	
2	6 (50.0)	2 (40.0)	
3	2 (16.7)	0 (0.0)	
Length of hospital admission, days	4 (4–5)	4 [3–5]	0.1140
Length of ICU admission, days	3 (2–3)	2 (2–3)	0.0677
New-onset heart failure	0 (0.0)	0 (0.0)	-
Stroke ≤ 30d	0 (0.0)	0 (0.0)	-
Death ≤ 30d	0 (0.0)	0 (0.0)	-

In the univariate analysis, all pre-operative and operative characteristics were examined individually to determine whether they affected the odds of a patient experiencing POAF. None of the variables were associated with POAF except HPMA treatment. Patients who had HPMAs placed showed an 83% reduction in POAF (OR = 0.17, *p* = 0.0248) compared to control patients who did not have HPMAs placed (Table 4). Multivariable analysis yielded a similar result (OR = 0.07, *p* = 0.0156) after controlling for all pre-operative characteristics that were found to be significantly different between groups (Table 5).

**Table 4:** – Univariate analysis on POAF.

Covariate	uOR (95% CI)	*p*-value
HPMA (Yes vs. No)	0.17 (0.04–0.79)	**0.0240**
*Pre-operative variables*		
Age	1.00 (0.94–1.06)	0.9399
Gender (Male vs. Female)	1.96 (0.58–6.67)	0.2807
EF	0.99 (0.91–1.08)	0.8810
STS	0.61 (0.23–1.65)	0.3324
Prior MI	1.19 (0.43–3.29)	0.7332
STEMI vs. NSTEMI	2.62 (0.15–47.17)	0.5128
Stenting (Yes vs. No)	1.31 (0.39–4.34)	0.6622
𝛽-Blocker (Yes vs. No)	0.68 (0.25–1.85)	0.4450
BMI	1.00 (0.89–1.12)	0.9768
Glomerular filtration rate	1.00 (0.98–1.04)	0.7522
Creatinine	1.25 (0.23–6.63)	0.7960
Pre-operative renal failure (Yes vs. No)	1.07 (0.43–2.67)	0.8823
Hypertension (Yes vs. No)	1.14 (0.36–3.66)	0.8222
Diabetes (Yes vs. No)	0.69 (0.24–1.96)	0.4838
*Operative variables*		
Number of vessels bypassed 4 (Ref)	Reference	
3	1.75 (0.33–9.31)	0.9707
2	1.25 (0.20–7.92)	0.9741
1	-	-
Cardiopulmonary bypass time	1.00 (0.98–1.02)	0.8465
Aortic cross-clamp time	1.00 (0.98–1.03)	0.7297

**Table 5:** – Multivariable analysis on POAF.

Covariate	aOR (95% CI)	*p*-value
HPMA (Yes vs. No)	0.07 (0.01–0.60)	**0.0156**
Age	1.01 (0.94–1.08)	0.7727
STS	0.29 (0.07–1.19)	0.0847
𝛽-Blocker (Yes vs. No)	1.31 (0.39–4.40)	0.6607

## Discussion

New-onset postoperative atrial fibrillation (POAF) is a significant concern for patients undergoing coronary artery bypass grafting surgery, with up to 40% of patients developing POAF. POAF confers an increased risk of inpatient mortality, extended admission duration, and risk of MACE. While POAF resolves in around 90% of cases by 8 weeks post-operatively, it remains a significant independent predictor of long-term mortality [8], and therefore a post-operative complication that there is great merit in addressing. While the most significant effect of POAF is of course the morbidity/mortality burden, it is also important to consider the economic impact of POAF, which can be significant, both for the patient and the healthcare system. AF management imposes huge financial burdens on health systems with an estimated annual direct cost of $26 billion in the USA [26].

Patients who develop POAF may require additional medical attention, including prolonged hospitalization due to additional complications, additional diagnostic tests, and medications. These additional interventions and support will increase the cost of care for the patient, provider, and insurer, resulting in an increased economic burden on the healthcare system. For patients who experience a stroke, a common complication associated with AFib, the U.S. average per patient per year cost was reported to be $59,900 [27]. For AKI, the increased index hospitalization cost was $38,358 ($77,178 for patients with AKI vs. $38,820 for those without) [28].

Patients with POAF may also require additional follow-up care, including outpatient visits, rehabilitation, monitoring, and an increase in hospital readmission which can further increase the economic impact of this condition. This study explored the potential of using aseptically processed human placental membrane allografts after CABG surgery to prevent POAF, one of the most common postoperative complications, contributing significantly to morbidity and mortality through thromboembolic events such as stroke as well as complications such as AKI. This study found a significant reduction in POAF in patients treated with HPMA, both in the initial analysis and also in univariate and multivariable analyses conducted to account for any differences in characteristics between groups. Due to the retrospective observational nature of this study, cohorts were not matched in demographics or clinical characteristics, though through our rigorous statistical analysis, the effects of any cohort differences were accounted for. There were no significant differences in operative characteristics between the HPMA-treated group and the control group.

The option of using HPMA was found to be safe with no significant differences in post-operative complications such as AKI, stroke, or death at thirty days. In this study, the HPMAs were placed strategically on the epicardial surface of the heart to cover key ventricular and atrial sites. The study hypothesized that the innate anti-inflammatory, anti-adhesive, and antimicrobial properties of placental tissue would offer benefits in terms of mitigation of inflammation, tissue protection, scar remodeling, and modulating the response of macrophages towards a favorable outcome. The significant decrease in POAF incidence from 35.2 to 8.3% with HPMA treatment suggests the use of HPMA to mitigate POAF is a promising approach that supports further clinical and scientific investigation. The study found that HPMA treatment did not affect the risk of AKI following the CABG procedure.

These results suggest that identifying patients who could benefit from HPMA before the CABG procedure is important for future investigation, to provide robust results that have even greater potential to change clinical practice than this work. T2-weighted cardiac MRI imaging is a well-established modality to evaluate the inflammatory burden of the myocardium and other tissues, by assessing edema and fluid distribution (pericardial edema in the case of new-onset post-operative atrial fibrillation). Previous work has also shown the association between POAF, and post-operative myocardial inflammation as evaluated by T2-weighted MRI, as well as the early success in humans of placing a human amniotic patch, resulting in reduced inflammation, pericardial edema, and freedom from AFib [29]. The present study suggests that placing HPMA on the heart topically can be a simple solution at the end of a coronary artery bypass grafting case that could save the morbidity and mortality risk of new-onset postoperative atrial fibrillation in patients with possible shorter ICU and total hospital admission.

Given that there is no consensus on a molecular pathway for the pathophysiology of AFib despite the clear link with myocardial inflammation, a precise understanding of the mechanism of the therapeutic benefit conferred by HPMA is unlikely at present. It has been suggested that the pro-inflammatory AGE pathway as well as the process of fibrosis are involved in the pathogenesis of AFib [30]. AGEs are formed by nonenzymatic glycation of proteins, lipids, and nucleic acids with reducing sugars, a process that occurs at significantly greater rates in metabolic syndromes such as diabetes mellitus. AGEs interact with the receptor (RAGE), to produce ROS, and consequently factors such as TNF-𝛼 and other pro-inflammatory cytokines, factors which are strongly implicated in AFib. Atrial structural remodeling due to fibrosis is also thought to be significant in AFib; both AGE and RAGE are implicated in atrial fibrosis. Data from both murine models, as well as human patients, suggests that the levels of AGEs in both plasma and tissue, specifically the atrium, are elevated in patients with AFib.

The preliminary hypothesis for the mode of action for the aseptically processed human placental membrane allograft evaluated in this study relies on the innate immunomodulatory and anti-inflammatory properties of placental tissues. Characterization of HPMAs has shown that naturally occurring anti-inflammatory properties are retained in the aseptically processed tissue [31]. By inhibiting pathways such as RAGE, and reducing ROS activity, we suggest that HPMA placement reduces post-operative myocardial and pericardial inflammation, thus reducing the incidence of POAF. Further in-vitro and in-vivo investigations are underway to elucidate actual molecular mechanisms providing the positive clinical outcome observed in this study. All patients in the study received LAAL, using the AtriClip device, but it was not taken into consideration when interpreting the results. Recently LAAL was found to be non-inferior to warfarin treatment for stroke prevention in patients with AFib acutely and over 2 years post-intervention [32, 33]. Healey and colleagues also concluded that LAAL was preferred, as over 90% of thrombi are found to originate from the LAA. Additionally, LAAL does not significantly increase procedure time and is not thought to increase the risk of post-operative complications including HF, POAF, or bleeding [34].

However, there is anecdotal and institutional experience that the material used to clip the LAA may incite a peri-clip inflammatory reaction contributing to the incidence of POAF. The study highlights the need to consider the potential effects of LAAL when evaluating the results of using HPMA. While occlusion of the LAA is not always routine practice in patients without a history of AFib, such as the patients in this study, it was deemed to be a clinically appropriate intervention in this case. In all patients in this study, there was a valid reason for LAAL. Each patient had one or more such reasons, including a CHA_2_DS_2_-VASc score ≥ 2 or on transesophageal echocardiography (TEE), an LAA velocity (LAAV) of ≤ 40 cm/s. Previous work has shown that a reduced LAAV is a strong predictor of LAA thrombus formation [35]. These criteria are similar to those used by the multi-national LeAAPS randomized controlled trial, which is currently underway, and aims to show the benefit of prophylactic left atrial appendage closure for stroke prevention [36]. Post-operatively, all subjects were placed on guideline-directed dual anti-platelet therapy (aspirin and clopidogrel) [37] as well as a 𝛽-blocker post-operatively to achieve blood pressure management with rate control. If POAF did occur, the first line of defense was amiodarone. Patients who developed POAF were followed by an electrophysiologist, with the potential for future catheter or hybrid ablation [38, 39] if POAF progressed to persistent AFib.

## Limitations

While the lack of totally matched cohorts was a limitation, our subsequent statistical analyses attempted to account for small demographic and clinical differences between the two groups. This was achieved through not only performing univariate but also multivariable analyses. These enabled our conclusions to consider the differences in patient populations, concluding that the only characteristic or treatment associated with a difference in POAF was the epicardial placement of HPMAs. It has been shown that in the regression-based approach utilized here is superior to group-matching in retrospective studies such as this, where group differences may exist [40]. While matching is often chosen to make groups appear comparable, a regression approach has in fact been shown to be much more effective at eliciting a treatment effect, hence why it was chosen in this study, sacrificing the appearance of demographically matched study cohorts.

Additionally, our relatively small sample size meant that certain differences between groups in specific outcomes (such as length of ICU admission), remained as trends that did not quite reach statistical significance. While we recognize such trends, it is impossible to make any conclusions from them until they reach statistical significance, which will only be possible with larger sample sizes. Nonetheless, we conducted this study as a pilot study to collect preliminary data and guide the design of a multi-center randomized controlled trial. The results of this study are encouraging and have been instrumental in designing the final study protocol for the trial, which is currently undergoing deployment.

We recognize that there is a very small chance that we may have missed a patient with a history of preoperative paroxysmal atrial fibrillation. All patients who were admitted as inpatients prior to their operation were on continuous EKG telemetry monitoring, and if AFib was detected, a confirmatory 12-lead EKG would be carried out. All patients who presented to the hospital on the day of surgery had previously had multiple EKGs in the process of preoperative clinical assessment and evaluation and at least one EKG carried out by their cardiologist’s office prior to referral for cardiac surgery -- often this would be a 24-hour monitoring EKG. They also underwent EKG testing in the cardiac surgery outpatient clinic, as well as the pre-operative assessment clinic. While we cannot exclude a patient who had paroxysmal AFib preoperatively, which wasn’t picked up by any of the multiple EKGs that were carried out, we are highly confident that this did not happen.

In conclusion, this study found that the application of three aseptically processed human placental membrane allografts on the surface of the heart after coronary artery bypass grafting was associated with a significant reduction in the incidence of new-onset postoperative atrial fibrillation and did not affect the risk of AKI. The study also found a decrease in inotrope use in patients who received the HPMA. The study suggests that the application of HPMA can be a safe and effective solution to reduce POAF in CABG patients without a history of heart failure, chronic kidney disease, or prior incidence of AFib. While encouraging, the results of this study warrant further prospective studies to confirm these first positive clinical outcomes and better understand the mechanism of action associated with HPMA placement.

## Conclusions

This study has shown that epicardial placement of aseptically processed human placental membrane allografts is a safe, simple, yet effective intervention that can be undertaken at the end of a CABG procedure. This study showed a significant 83% reduction in POAF incidence, even after accounting for differences in pre-existing demographic and clinical characteristics through undertaking univariate and multivariable analysis. While exact molecular and cellular mechanisms for the pathophysiology of AFib and POAF are not widely agreed upon, it is difficult to explain the positive impact of the HPMAs. However, given the known anti-inflammatory properties of placental tissues, as well as the implication of inflammatory pathways such as RAGE in AFib, we propose that HPMA placement may be associated with a reduction in post-operative cardiac inflammation and therefore provides a novel approach to reduce the risk of POAF, thus potentially reducing ICU and hospital admissions, while improving long-term outcomes. While the retrospective nature of this study and relatively small sample size were limitations, this study was a pilot study, with the aim of providing us with preliminary data to guide a forthcoming multi-center randomized controlled trial. This study provided encouraging results that have been instrumental in developing our randomized trial, which is currently in the process of deployment. We look forward to sharing the results from this forthcoming study in due course.

## Data Availability

Key data generated or analyzed during this study are included in this published article and its supplementary information files, with all data available on reasonable request to the corresponding author, Dr Zain Khalpey.

## Abbreviations

AFib: Atrial fibrillation
AGE: Advanced glycation end products
AHA: American heart association
AKI: Acute kidney injury
BMI: Body mass index
CABG: Coronary artery bypass grafting
CKD: Chronic kidney disease
CPB: Cardiopulmonary bypass
HF: Heart failure
HPMA: Aseptically processed human placental membrane allograft
ICU: Intensive care unit
KDIGO: Kidney Disease: Improving Global Outcomes
LAA: Left atrial appendage
LAAL: Left atrial appendage ligation
LVEF: Left ventricular ejection fraction
MACE: Major adverse cardiovascular events
MI: Myocardial infarction
POAF: New–onset postoperative atrial fibrillation
ROS: Reactive oxygen species
STS: Society of thoracic surgeons
TEE: Transesophageal echocardiograph
XCT: Aortic cross–clamp time
