# Correction: Epicardial placement of human placental membrane allografts in coronary artery bypass graft surgery is associated with reduced postoperative atrial fibrillation: a pilot study for a future multi-center randomized controlled trial

**DOI:** 10.1186/s13019-024-02942-1

**Published:** 2024-07-01

**Authors:** Zain Khalpey, Ujjawal Kumar, Pamela Hitscherich, Usman Aslam, Evangelia Chnari, Marc Long

**Affiliations:** 1 Department of Cardiothoracic Surgery, Heart and Vascular Institute, 10210 N 92nd St Suite 300, HonorHealth, Scottsdale, AZ 85258 USA; 2 Gonville & Caius College, University of Cambridge, Trinity Street, Cambridge, CB2 1TA UK; 3 MTF Biologics, 125 May Street, Edison, NJ 08837 USA; 4 General Surgery Residency Program, HonorHealth, Phoenix, AZ 85250 USA

**Journal of Cardiothoracic Surgery (2024) 19:315**

10.1186/s13019-024-02822-8

Following the publication of the original article [1], the DOI for reference 23 was missing and should have been included as 10.7759/cureus.59876.

Also, the ORCiD 0009-0006-2607-5555 (Zain Khalpey) and 0000-0001-8655-5856 (Ujjawal Kumar) for the authors have been included.

Also the authors Zain Khalpey and Ujjawal Kumar were denoted as equally contributing authors.

The original article has been corrected.
